# Case Report: Pulmonary sarcomatoid carcinoma complicating TP53 mutation treated successfully with Tislelizumab combined with Anlotinib—a case report

**DOI:** 10.3389/fgene.2022.949989

**Published:** 2022-07-22

**Authors:** Yu-Feng Li, Xin-Fei Zhao, Yue Tian, Xin-Yao Xiao, Cai-Yun Yan, Hua Shen

**Affiliations:** Department of Medical Oncology, The Affiliated Sir Run Run Hospital of Nanjing Medical University, Nanjing, China

**Keywords:** Tislelizumab, Anlotinib, pulmonary sarcomatoid carcinoma, TP53 mutation, hepatitis C, case report

## Abstract

Pulmonary sarcomatoid carcinoma (PSC) is a rare subtype of lung malignant tumor. Conventional chemotherapy has a suboptimal effectiveness. PSC has the characteristics of rapid disease progression and poor prognosis. We herein report a 56-year-old male patient with substantial smoking history was pathologically diagnosed as PSC, cT4N0M0 IIIA stage. Peripheral blood NGS showed TP53 mutation. The patient had poor tolerance to the first-line chemotherapy regimen “albumin paclitaxel + cisplatin,” but the severe anemia was significantly improved after 5 days of anti-angiogenic therapy with Anlotinib. At this time, the patient received anti-PD-1 immunotherapy with Tislelizumab. Half a month later, degree III liver injury occurred repeatedly. After excluding drug-induced liver injury, we found that HCV-RNA 3.10 × 10^5^ IU/ml and suspended all anti-tumor therapy. After the start of anti-HCV treatment with Epclusa, the treatment of Tislelizumab combined with Anlotinib was restarted, and there was no liver injury after that. The patient received monthly maintenance therapy with Tislelizumab combined with Anlotinib to the present. The pulmonary lesions continued to decrease, and only one lung cavity is left. The patient has achieved clinical complete remission (CCR) with PSF over 20 months. Our findings suggest that Tislelizumab combined with Anlotinib may be a preferred strategy in PSC complicating TP53 mutation. Core tip: Immune-check point inhibitors (ICIs) have been reported for the treatment of PSC in a small number of case reports and retrospective analysis, but there are few reports of ICIs combined with anti-angiogenic drugs. This patient was diagnosed as locally advanced PSC complicated with TP53 mutation and hepatitis C. After 14 cycles of Tislelizumab combined with Anlotinib treatment (during the course of treatment, several courses were not treated on time for economic reasons, rather than adverse reactions), the patient has achieved CCR. III degree liver injury occurred during the treatment, and the liver function returned to normal range after anti-hepatitis C treatment, which did not affect the continued treatment of this regimen.

## Introduction

Pulmonary sarcomatoid carcinoma (PSC) is a rare kind of lung malignant tumor, and its incidence accounts for about 0.1–0.4% of the total number of lung malignant tumors. It is common in elderly men with a large history of smoking (Mehrad et al., 2018). It features high degree of malignancy, rapid disease progression, and poor prognosis (Shum et al., 2016). Radical surgery can be selected for early PSC, but most patients miss the optimal time of surgery because of the late diagnosis (Tulsi et al., 2021). PSC shows insensitivity to traditional radiotherapy and chemotherapy (Gu et al., 2015).

With the wide application of next-generation sequencing (NGS), targeted therapy, and immunotherapy (Wang et al., 2021) in recent years, there are more options for the treatment of PSC (Li X. et al., 2020). A Chinese NGS study of 32 PSC patients showed that TP53 (69%) was the most common mutated tumor suppressor gene (Liang et al., 2019). The tumor protein TP53 gene, encoding the cellular tumor antigen p53, is the single most frequently mutated gene in human cancers (Vaddavalli and Schumacher, 2022). However, there are no clinically approved drugs targeting TP53 mutations currently (Ku et al., 2017). In addition, Nalan A. Babacan’s summary analysis showed that nearly 90% of PSC patients had PD-L1 ≥ 1%, and the level of PD-L1 expression was significantly correlated with therapeutic effect of immune-check point inhibitors (ICIs) (Babacan et al., 2020). The therapeutic effect of selective application of ICIs in patients with related tests is much better than that of traditional radiotherapy and chemotherapy (Li X. et al., 2020). However, due to the low incidence of PSC, the application of ICIs is mostly reported in case reports, retrospective summary analysis, and small sample stage II clinical trials. Further attention needs to be paid to its adverse reactions, combination treatments, treatment course, and so on.

We herein report a case of advanced PSC treated with first-line conventional chemotherapy (albumin paclitaxel + cisplatin) and then adjusted to Anlotinib combined with Tislelizumab, which is the first case reporting the effectiveness of Anlotinib combined with Tislelizumab in the treatment of PSC complicating TP53 mutation.

## Case report

A 56-year-old man with a history of 30 pack-years smoking and drug use complained of chest tightness, asthma for 1 week. A physical examination revealed the following: ECOG 4 score, height 176 cm, weight 46 kg. The conjunctiva of the mouth and lower eyelid was pale.

A laboratory examination revealed the following: White blood cell count 64.9 × 10^9^/L, neutrophil absolute value 59.95 × 10^9^/L, hemoglobin 57 g/L, platelet count 377 × 10^9^/L, HBsAb 153.2mIU/ml, HBcAb 8.52S/CO, HCV-Ab 9.29S/CO. There was no obvious abnormality in quantitative detection of hepatitis B DNA, liver function and autoimmune antibody.

Chest CT showed that the left lung occupied a large space, with a size of about 13.2*11.1 cm, uneven density, local invasion of adjacent bronchial branches, narrowing and occlusion of adjacent bronchial branches, and interstitial changes of the left lung.

**FIGURE 1:**
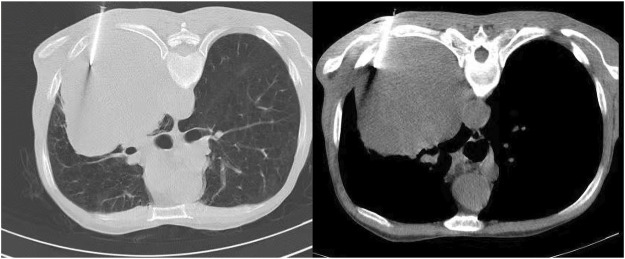
CT-guided percutaneous lung biopsy (CT-PTNB). CT = computed tomography.

**FIGURE 2:**
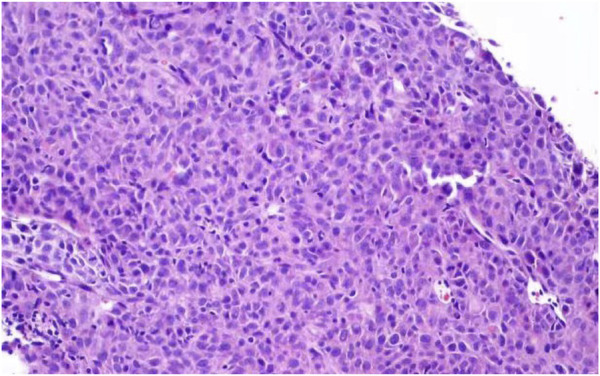
Microscopic findings of lung biopsy specimens. Routine biopsy showed: poorly differentiated adenocarcinoma. Immunohistochemistry showed: tumor cells Vimentin (+), Ki67 (about 50%+), CK-pan (scattered weak +), CK-L (+), CKH (−), CK7 (scattered weak+), NapsinA (−), TTF1 (−), CK5/6 (−), P63 (−), P40 (−), Syn (−), EMA (−), CD34 (−), Desmin (−), S100 (−), MelanA (−), FLI1 (−), SOX10 (−), INI-1 (scattered weak +), and tended to sarcomatoid carcinoma.

Under CT guidance, the tip was determined to be located in the lesion (Figure 1). A 18G cutting needle was inserted, four fish-like tissues were cut, and the specimens were fixed with formalin. The biopsy pathology of the left lung mass showed poorly differentiated carcinoma. Immunohistochemistry showed: tumor cells Vimentin (+), Ki67 (about 50%+), CK-pan (scattered weak +), CK-L (+), CKH (−), CK7 (scattered weak +), NapsinA (−), TTF1 (−), CK5/6 (−), P63 (−), P40 (−), Syn (−), EMA (−), CD34 (−), Desmin (−), S100 (−), MelanA (−), FLI1 (−), SOX10 (−), INI-1 (scattered weak +), and tended to sarcomatoid carcinoma (Figure 2). We performed targeted enrichment using a GenCap Custom Exome Enrichment kit (MyGenostics, Beijing, China) for the patient’s peripheral blood. This enrichment kit contains 562 tumor-driven genes, 45 chemotherapy genes, 90 genetic risk-related genes, and 35 HRR-related genes (Supplementary Table S1). The results of peripheral blood NGS (including 595 tumor-related genes) showed mutation in exon 4 of TP53 (TP53 p.T125K mutation); TMB was 7.7 mutations/Mb, moderate TMB; MSS was stable; and MMR gene had no mutation. According to the patient’s will, we did not perform PD-L1 test. And tumor tissue NGS could not be performed because of limited biopsy tissue. Therefore, the patient was diagnosed with sarcomatoid carcinoma of the left lung in stage IIIA (cT4N0M0).

The patient received multiple red blood cell suspension transfusions and parenteral nutrition support. In September 2020, he was treated with albumin paclitaxel (150 mg, d1/d8) combined with cisplatin (20 mg, d1-3). IV degree myelosuppression followed, and the treatment was abandoned in the second half of the cycle. After 5 days of oral administration of Anlotinib (12 mg qd), the general condition of the patient was significantly improved and he did not need to rely on blood transfusion. He received 200 mg immunotherapy with Tislelizumab on September 29.

Half a month later, the patient found III degree liver injury, accompanied by positive antinuclear antibody. After the recovery of liver function, patients were treated with albumin paclitaxel (150 mg D1) combined with cisplatin (20 mg D1-3) and Anlotinib on October 23. II degree myelosuppression occurred in the follow-up.

The III degree liver injury was found again in November 2020, and the autoantibodies were all negative. CT evaluation was PR. After the recovery of liver function, patients were treated with Anlotinib combined with Tislelizumab for the second cycle on November 24.

Unfortunately, II degree liver injury occurred again on December 22, and the HCV-RNA of hepatitis C was 3.10 × 10^5^ IU/ml, so we suspended all antineoplastic therapy. However, the quantity of HCV-RNA was negative on 6 January 2021.

**FIGURE 3:**
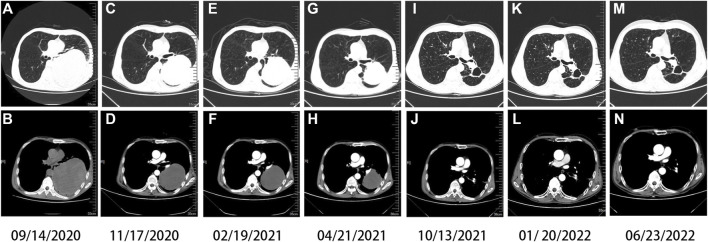
Chest computed tomography (CT). Imaging findings during the patient’s course **(A,B)** at baseline (solid tumor, dmax = 13.2 cm × 11.1 cm), **(C,D)** after one cycle of Tislelizumab and two cycles of chemotherapy (solid tumor, dmax = 11 cm × 8.7 cm), **(E,F)** after three cycles of Tislelizumab (solid tumor, dmax = 9.5 cm × 8.1 cm), **(G,H)** after five cycles of Tislelizumab (solid tumor, dmax = 7.4 cm × 7.0 cm), **(I,J)** after ten cycles of Tislelizumab (cavity, dmax = 3.9 cm × 3.2 cm), and **(K,L)** after thirteen cycles of Tislelizumab (cavity, dmax = 3.6 cm × 2.8 cm); **(M,N)** in recent recheck (cavity, dmax = 3.3 cm × 2.5 cm), Anlotinib is in continuous use.

On 20 January 2021, he began to receive antiviral therapy with sufosbuvir and velpatasvir tablets (Epclusa), and was treated with Anlotinib combined with Tislelizumab for the third cycle. There was no liver injury in the follow-up, and the patient was treated with Anlotinib combined with Tislelizumab every month (Figure 3). His last reexamination was on 23 June 2022. A physical examination revealed the following: ECOG 1 score, weight 50 kg. There was no obvious abnormality in tumor index, blood routine, and biochemical index. CT shows that the cavity on the lung shrinks again.

According to the patient’s recovery condition, conversion therapy can be considered at present. However, he is unwilling to undergo surgical treatment, thus we keep him on maintenance medication and closely monitor adverse reactions.

## Discussion

At present, ICIS targeting PD-1/PD-L1 has become an indispensable part of the treatment of non-small cell lung cancer (NSCLC), and the expression level of PD-L1 is a common marker for predicting the efficacy of immunotherapy (Doroshow et al., 2019). Previous studies have shown that PD-L1 is highly expressed in PSC. Velchet reported that 69.2% (9/13) of the patients were positive for PD-L1 (Velcheti et al., 2013), and 36.5% (54/148) of the patients were positive in the Yang Z study (Yang et al., 2019). Charlotte Domblides’s retrospective evaluation of the efficacy of second- and third-line ICI treatment in 37 patients with PSC showed that regardless of the expression level of PD-L1, the ORR of patients after immunotherapy was 40.5%, the DCR was 64.8%, and the median OS was 12.7 months (Domblides et al., 2020). Patients with PSC who only received routine chemotherapy had 5–7.7 months of OS and 0–16.5% of ORR (Vieira et al., 2013; Bonomi et al., 2019). These data show that immunotherapy is effective in patients with PSC.

The tumor suppressor protein p53, a transcription product of the anti-oncogene TP53, is a critical factor in preventing cellular cancerization and killing cancer cells by inducing apoptosis. As a result, p53 is often referred to as the “guardian of the genome” (Hibino and Hiroaki, 2022). Mutation of the TP53 tumor suppressor gene is the most common genetic alteration in cancer, and almost 1,000 alleles have been identified in human tumors. Virtually all TP53 mutations are thought to compromise wild-type p53 activity, thus producing mutant p53 protein, which results in the dysfunction of wild-type p53 and promotes malignant transformation of cells (Kennedy and Lowe, 2022). Lysine(K) residue acetylation is a critical epigenetic modification to influence protein structure and gene expression (Verdone et al., 2006). On the DBD (central deoxyribonucleic acid (DNA)-binding domain)of p53, there is an important acetylation site K120, which is catalyzed by three members (Tip60, MOF, and MOZ) of the MYST HAT family (Liu et al., 2019). Tip60 and MOF acetylates p53 at K120 to induce the expression of proapoptotic genes (like PUMA and Bax) (Li et al., 2009; Liu et al., 2019). MOZ-mediated K120 acetylation of p53 specifically enhances its antiproliferative activity. In cancers, K120 is often mutated. The tumor-derived mutant p53 (K120R) is defective for Tip60-mediated acetylation, thus abrogating p53-dependent activation of apoptosis but having no significant effect on cell growth arrest (Li et al., 2009). In addition to K120 acetylation, the multimodular structure of p53 makes it a perfect platform to undergo a multitude of covalent modifications. Actually, in most cases, there is widespread crosstalk between modifications (Gu and Zhu, 2012). In this case, our patient’s mutation occurred at amino acid 125, which is close to the K120 site, and there may be nearby modification crosstalk (one modification affects another in its local area). Therefore, the mutation at amino acid 125 may produce similar results as the K120 mutation, accordingly preventing the activation of apoptosis and promoting cancer progression.

Preclinical studies have shown that patients with TP53 mutations may benefit from Wee1 inhibitor AZD1775 (Ku et al., 2017). However, no targeted therapy for TP53 has been approved. Fortunately, studies have shown that TP53 mutation can significantly activate immune-checkpoints, initiate effector T cells, and increase immune factors expression levels. In lung cancer immunotherapy, TP53 mutation may be a positive predictor of immunotherapy (Cui et al., 2021). What’s more, studies have shown that smokers are more likely to respond to ICI treatment (Li J. J. N. et al., 2020). Based on these evidences, we chose anti-PD-1 immunotherapy.

Tislelizumab is a humanized IgG4 monoclonal antibody against PD-1, which is different from other PD-1 antibodies in that it can avoid binding to Fc receptors on macrophages through unique modification of the Fc segment, thus eliminating the ADCP effect and avoiding the anti-tumor effect due to the decrease of the number of T cells (Liu and Wu, 2020). In lung cancer, it has been approved by the National Medical Products Administration (NMPA) for combined chemotherapy in the treatment of advanced NSCLC. It has been reported that PD-1 inhibitors such as Nivolumab, Pembrolizumab, and Toripalimab have been used in the treatment of PSC (Cimpeanu et al., 2020; Jin and Yang, 2020; Jiao et al., 2021). Here, we report for the first time the use of Tislelizumab in the treatment of advanced PSC, and the tumor retraction has reached almost complete remission.

The patient had a history of injection drug use, and the baseline showed that HBV-DNA was negative and HCV-Ab 9.29S/CO↑. Before anti-tumor therapy, the screening rate of hepatitis C was low (Hwang et al., 2014; Mohamed et al., 2020). The baseline liver function of this patient was not abnormal, and he denied the history of hepatitis, so no HCV-RNA test was performed. It has been reported that HCV reactivation can occur after chemotherapy (Talima et al., 2019; Xu et al., 2020), but HCV infection has no adverse effect on chemotherapy patients in general (Liu et al., 2017). There are few studies on the effect of HCV on the treatment of ICI, and the reactivation of HCV during ICI treatment is rare (Pertejo-Fernandez et al., 2020; Alkrekshi and Tamaskar, 2021). A retrospective study by Professor Zhang showed that 6 of the 114 HBsAg (+) cancer patients developed viral reactivation after receiving ICI treatment (5.3%). Prophylactic antiviral therapy significantly reduced the risk of viral reactivation (1.2 vs.17.2%, *p* = 0.004) and reduced the incidence of HBV-related hepatitis (1.2 vs.13.8%, *p* = 0.019) (Zhang et al., 2019). It has been reported that a 55-year-old male liver cancer patient with baseline HCV- RNA 152 × 105 IU/ml showed elevated transaminase and second-degree liver injury after the first Nivolumab treatment. The liver injury was relieved after prednisone treatment, and the subsequent use of Nivolumab was not affected (Alkrekshi and Tamaskar, 2021). Therefore, in patients with chemotherapy and immunotherapy, attention should be paid to the reexamination of HBV-DNA and HCV-RNA when liver injury occurs (Gomes et al., 2020).

It has been reported that patients with PSC tried antivascular therapy (Jin and Yang, 2020; Kong et al., 2020). This patient had severe anemia at the time of first admission, and it was difficult to improve by RBC transfusion. After the use of Anlotinib, the anemia was significantly improved, which won the opportunity for subsequent treatment. Anlotinib is a novel multi-target tyrosine kinase inhibitor, which mainly targets VEGFR, EGFR and PDGFR to inhibit tumor angiogenesis. Anlotinib has been approved for third-line treatment of NSCLC, SCLC, second-line treatment of soft tissue sarcoma, and medullary thyroid carcinoma (Syed, 2018; Gao et al., 2020). In our case, the patient was well tolerated with Anlotinib, and no adverse side effects were observed in the follow-ups.

Anti-angiogenic drugs combined with ICI can be synergistic. Anti-angiogenic drugs improve tumor microenvironment by resisting tumor angiogenesis, while anti-PD1 immunotherapy can activate immune cells and promote vascular normalization, both of which form a positive feedback circulatory mechanism. Therefore, anti-angiogenic therapy combined with immunotherapy can act synergistically with tumor cells and improve the curative effect (Huang et al., 2018). After the patient gave up chemotherapy, the patient was treated with Anlotinib combined with Tislelizumab to the present.

As far as we know, this is the first report showing that Tislelizumab combined with Anlotinib is effective in the treatment of PSC complicating TP53 mutation, and this is one of the few reported cases of chemotherapy and immunotherapy in tumor patients with HBV and HCV infection. Immunotherapy combined with anti-angiogenic drugs may be a potential and promising strategy for the treatment of PSC, but its effectiveness and safety need to be further verified in more cases, and more accurate populations need to be selected. In the future, we will continue to follow up the patient to observe the adverse events of ICI and related drug resistance. Moreover, in a variety of clinical cases, we will strive to select more accurate people for accurate treatment.

## Data Availability

The original contributions presented in the study are included in the article/Supplementary Material, further inquiries can be directed to the corresponding authors.
