# Supplementary material for: Case Report: Pulmonary sarcomatoid carcinoma complicating TP53 mutation treated successfully with Tislelizumab combined with Anlotinib—a case report
Source: Front Genet. 2022 Jul 22;13:949989. doi: 10.3389/fgene.2022.949989 (PMC9355298; doi:10.3389/fgene.2022.949989)
Supplement: Supplementary file 1 [file Table1.pdf]

Table1:

562 tumor-driven genes:

ABL1	ABL2	ABRAXAS1	ACVR1	ACVR1B	ADGRA2	AGO2	AKT1
AKT2	AKT3	ALK	ALOX12B	AMER1	ANKRD11	APC	AR
ARAF	ARFRP1	ARID1A	ARID1B	ARID2	ARID5B	ASXL1	ASXL2
ATM	ATR	ATRX	AURKA	AURKB	AXIN1	AXIN2	AXL
B2M	BABAM1	BAP1	BARD1	BBC3	BCL10	BCL2	BCL2L1
BCL2L11	BCL2L2	BCL6	BCOR	BCORL1	BCR	BIRC3	BLM
BMPR1A	BRAF	BRCA1	BRCA2	BRD4	BRIP1	BTG1	BTG2
BTK	CALR	CARD11	CARM1	CASP8	CBFB	CBL	CCND1
CCND2	CCND3	CCNE1	CCNQ	CD22	CD274	CD276	LMO1
LRP1B	LTK	LYN	LZTR1	MAF	MAGI2	MALT1	MAP2K1
MAP2K2	MAP2K4	MAP3K1	MAP3K13	MAP3K14	MAPK1	MAPK3	MAPKAP1
MAX	MCL1	MDC1	MDM2	MDM4	MED12	MEF2B	MEN1
MERTK	MET	MGA	MITF	MKNK1	MLH1	MPL	MRE11
MSH2	MSH3	MSH6	MSI1	MSI2	MST1	MST1R	MTAP
MTOR	MUTYH	MYB	MYBL1	MYC	MYCL	MYCN	MYD88
MYOD1	NBN	NCOA3	NCOR1	NEGR1	NF1	NF2	NFE2L2
NFIB	NFKBIA	NKX2-1	NKX3-1	NOTCH1	NOTCH2	NOTCH3	NOTCH4
NPM1	NRAS	NSD1	NSD2	NSD3	NT5C2	ERBB3	ERBB4
ERCC2	ERCC3	ERCC4	ERCC5	ERF	ERG	ERRFI1	ESR1
ETV1	ETV4	ETV5	ETV6	EWSR1	EZH1	EZH2	EZR
TENT5C	FANCA	FANCC	FANCD2	FANCE	FANCF	FANCG	FANCL
FAS	FAT1	FBXW7	FGF10	FGF12	FGF14	FGF19	FGF23
FGF3	FGF4	FGF6	FGFR1	FGFR2	FGFR3	FGFR4	FH

FLCN	FLT1	FLT3	FLT4	FOXA1	FOXL2	FOXO1	FOXP1
FRS2	FUBP1	FYN	GABRA6	GATA1	GATA2	GATA3	GATA4
GATA6	GID4	GLI1	GNA11	GNA13	GNAQ	GNAS	GPS2
GREM1	GRIN2A	GRM3	GSK3B	H3-3A	RAC2	RAD21	RAD50
RAD51	RAD51B	RAD51C	RAD51D	RAD52	RAD54L	RAF1	RANBP2
RARA	RASA1	RB1	RBM10	RECQL	RECQL4	REL	RET
COP1	RHEB	RHOA	RICTOR	RIT1	RNF43	ROS1	RPS6KA4
RPS6KB2	RPTOR	RRAGC	RRAS	RRAS2	RSPO2	RTEL1	RUNX1
RUNX1T1	RXRA	RYBP	SDC4	SDHA	SDHAF2	SDHB	SDHC
SDHD	SESN1	SESN2	SESN3	SETD2	SF3B1	SGK1	SH2B3
SH2D1A	SHOC2	SHQ1	SLC34A2	SLIT2	SLX4	SMAD2	SMAD3
SMAD4	SMARCA2	SMARCA4	SMARCB1	SMARCD1	SMO	SMYD3	SNCAIP
SOCS1	SOS1	SOX10	SOX17	CD70	CD74	CD79A	CD79B
CDC42	CDC73	CDH1	CDK12	CDK4	CDK6	CDK8	CDKN1A
CDKN1B	CDKN2A	CDKN2B	CDKN2C	CEBPA	CENPA	CHD2	CHD4
CHEK1	CHEK2	CIC	CREBBP	CRKL	CRLF2	CSDE1	CSF1R
CSF3R	CTCF	CTLA4	CTNNA1	CTNNB1	CUL3	CUL4A	CXCR4
CYLD	CYP17A1	CYSLTR2	DAXX	DCUN1D1	DDR1	DDR2	DICER1
DIS3	DNAJB1	DNMT1	DNMT3A	DNMT3B	DOT1L	DROSHA	DUSP4
E2F3	EED	EGFL7	EGFR	EIF1AX	EIF4A2	EIF4E	ELF3
ELOC	EMSY	EP300	EPAS1	EPCAM	EPHA3	EPHA5	EPHA7
EPHB1	EPHB4	ERBB2	NTHL1	NTRK1	NTRK2	NTRK3	NUF2
NUP93	NUTM1	P2RY8	PAK1	PAK3	PAK5	PALB2	PARP1
PARP2	PARP3	PAX5	PAX8	PBRM1	PDCD1	PDCD1LG2	PDGFRA
PDGFRB	PDK1	PDPK1	PGR	PHOX2B	PIK3C2B	PIK3C2G	PIK3C3
PIK3CA	PIK3CB	PIK3CD	PIK3CG	PIK3R1	PIK3R2	PIK3R3	PIM1

PLCG2	PLK2	PMAIP1	PMS1	PMS2	PNRC1	POLD1	POLE
PPARG	PPM1D	PPP2R1A	PPP2R2A	PPP4R2	PPP6C	PRDM1	PRDM14
PREX2	PRKAR1A	PRKCI	PRKD1	PRKDC	PRKN	PRSS8	PTCH1
PTEN	PTP4A1	PTPN11	PTPRD	PTPRO	PTPRS	PTPRT	QKI
RAB35	RAC1	H3-3B	H3-5	HDAC1	HGF	H1-2	H2BC5
H3C1	H3C2	H3C3	H3C4	H3C6	H3C7	H3C8	H3C10
H3C11	H3C12	H3C14	H3C13	H3-4	HLA-A	HLA-B	HNF1A
HOXB13	HRAS	HSD3B1	HSP90AA1	ICOSLG	ID3	IDH1	IDH2
IFNGR1	IGF1	IGF1R	IGF2	IKBKE	IKZF1	IL10	IL7R
INHA	INHBA	INPP4A	INPP4B	INPPL1	INSR	IRF2	IRF4
IRS1	IRS2	JAK1	JAK2	JAK3	JUN	KAT6A	KDM5A
KDM5C	KDM6A	KDR	KEAP1	KEL	KIT	KLF4	KLHL6
KMT2A	KMT2B	KMT2C	KMT2D	KMT5A	KNSTRN	KRAS	LATS1
LATS2	SOX2	SOX9	SPEN	SPOP	SPRED1	SPTA1	SRC
SRSF2	STAG2	STAT3	STAT4	STAT5A	STAT5B	STK11	STK19
STK40	SUFU	SUZ12	SYK	TAF1	TAP1	TAP2	TBX3
TCF3	TCF7L2	TEK	TERC	TERT	TET1	TET2	TGFBR1
TGFBR2	TIPARP	TMEM127	TMPRSS2	TNFAIP3	TNFRSF14	TOP1	TOP2A
TP53	TP53BP1	TP63	TRAF2	TRAF7	TSC1	TSC2	TSHR
TYRO3	U2AF1	UPF1	VEGFA	VHL	VTCN1	CCN6	WT1
WWTR1	XIAP	XPO1	XRCC2	YAP1	YES1	ZBTB2	ZFHX3
ZNF217	ZNF703						

45 chemotherapy genes：

ABCB1	ABCC1	ABCC2	ABCC4	ABCG2	CCND1	CD3EAP	ESR1
ESR2	FCGR3A	FGFR4	GATA3	GGH	SOD2	TOP1	TP53

TPMT	TYMS	UGT1A1	CYP2D6	CYP3A4	CYP3A5	DPYD	ERCC1
ERCC2	PIK3CA	PTEN	PTGS2	SLC19A1	SLC22A2	SLCO1B3	COMT
CTNNB1	CYP2B6	CYP2C19	CYP2C8	GSTM3	GSTP1	LRP2	MTHFR
NRAS	NOS3	UGT1A7	UMPS	VEGFA			

90 genetic risk-related genes：

AKT1	ALK	APC	ATM	ATR	AXIN2	BAP1	BARD1
BLM	BMPR1A	BRCA1	BRCA2	EPCAM	ERCC2	ERCC3	ERCC4
ERCC5	FANCA	FANCC	FANCD2	FANCE	FANCF	FANCG	FH
MPL	MSH2	MSH6	MUTYH	NBN	NF1	NF2	NTRK1
PALB2	PDGFRA	PHOX2B	PIK3CA	RET	SDHA	SDHAF2	SDHB
SDHC	SDHD	SMAD4	SMARCB1	STK11	SUFU	TMEM127	TP53
BRIP1	CDC73	CDH1	CDK4	CDKN1B	CDKN2A	CHEK1	CHEK2
CTNNA1	CYLD	DICER1	EGFR	FLCN	GREM1	HNF1A	HOXB13
HRAS	KIT	LMO1	MAX	MEN1	MET	MITF	MLH1
PMS1	PMS2	POLD1	POLE	PRKAR1A	PTCH1	PTEN	RAD50
RAD51C	RAD51D	RB1	RECQL4	TP53BP1	TSC1	TSC2	TSHR
VHL	WT1						

35 HRR-related genes：

ATM	ATR	BAP1	BARD1	BLM	BRCA1	BRCA2	BRIP1
CDH1	CDK12	CHEK1	CHEK2	ERCC1	FANCA	FANCC	FANCD2
FANCE	FANCF	FANCL	MRE11	NBN	PALB2	POLD1	POLE
PRKDC	RAD50	RAD51B	RAD51C	RAD51D	RAD52	RAD54L	SLX4
TP53	TP53BP1	XRCC2					
